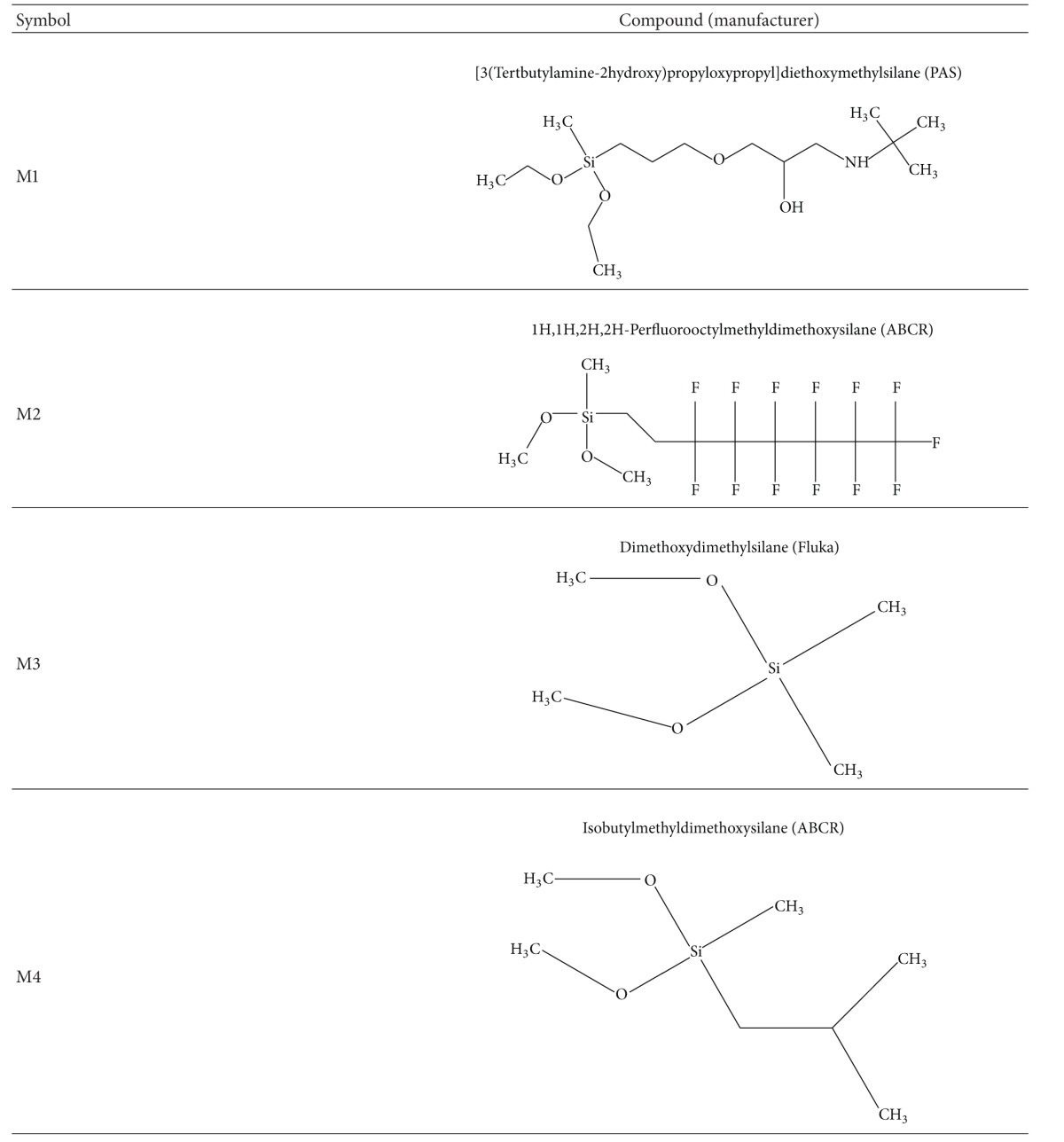# Effect of Plasma Processing and Organosilane Modifications of Polyethylene on *Aeromonas hydrophila* Biofilm Formation

**DOI:** 10.1155/2014/232514

**Published:** 2014-01-30

**Authors:** Dorota Kregiel, Kamila Niedzielska

**Affiliations:** Institute of Fermentation Technology and Microbiology, Lodz University of Technology, Ulica Wolczanska 171/173, 90-924 Lodz, Poland

## Abstract

The aim of our research was to study how the modifications of polyethylene—a material commonly used in medicine and water industry—influence bacterial cell attachment and biofilm formation. The native surface was activated and modified using two-step process consisting in the activation of native surface with a H_2_O vapor plasma followed by its treatment with various organosilanes, namely, [3(tertbutylamine-2hydroxy) propyloxypropyl] diethoxymethylsilane, 1H,1H,2H,2H-perfluorooctylmethyldimethoxysilane, dimethoxydimethylsilane, and isobutylmethyldimethoxysilane. The effect of polyethylene modification after chemical treatment was analyzed using surface tension measurement. The adhesive properties of *Aeromonas hydrophila* LOCK0968 were studied in water with a low concentration of organic compounds, using luminometric and microscopic methods, and the viability of the adhered bacterial cells was evaluated using the colony forming units method. After two-week incubation the chemically modified materials exhibited better antiadhesive and antibacterial characteristics in comparison to the native surface. Among the examined modifying agents, dimethoxydimethylsilane showed the best desired properties.

## 1. Introduction

*Aeromonas hydrophila* is the opportunist human pathogen which is widely distributed in aquatic environments [1]. Infections are typically acquired through two routes, either through ingestion or through exposure of open wounds. The primary clinical diseases from *Aeromonas* infections are gastroenteritis and bacterial septicemia. *Aeromonas*-related gastroenteritis is generally a self-limiting watery diarrhea lasting a few days to a few weeks. In individuals with weakened or impaired immune systems, this diarrhea can be chronic and severe (meaning a significant loss of water from the body). *Aeromonas* septicemia (a serious condition where bacteria are present throughout the body) generally occurs from wound infections or from gastroenteritis in individuals with weakened immune systems. It appears that all people are susceptible to gastroenteritis caused by *A. hydrophila*, although it is most frequently observed in young children (under 5 years of age) and older adults (over 60 years of age) [2–6]. Adhesion abilities of *A. hydrophila* are important in the pathogenesis.

Cell adhesion is a complex process, influenced by various physical and chemical properties of microorganisms, media, and surfaces. Aeromonads are able to form biofilms on both biotic and abiotic surfaces [5–8]. Knowledge of the factors involved in biofilm formation by *A. hydrophila* on inert surfaces is limited, but it has been shown that exopolysaccharides and swimming speed promote biofilm formation [7–9]. According to the literature, adhesion of *A. hydrophila *cells to different surfaces is usually correlated with the cell starvation. Even the small amounts of glucose may significantly impair both quorum sensing and biofilm formation [10]. These bacteria possess regulatory quorum-sensing system based on N-acyl homoserine lactone that is necessary for formation of microcolony structures [10–14]. Among different factors, the properties of solid surface seem to be artificially changed in order to prevent biofouling in natural water environments [15–19].

In physics and chemistry, plasma is a state of matter similar to gas in which a certain portion of the particles is ionized. It is typically obtained when gases are excited into energetic states by radiofrequency, microwave, or electrons from a hot filament discharge. The high density of ionized and excited species in the plasma can change the surface properties of normally inert materials. In particular, modification of the surface energetics of the materials can improve the adhesion strength and surface and coating properties. The application of plasma-based techniques is quite diverse, and examples of applications may include cleaning/sterilization or coating or deposition of a substrate. In effect, it can produce highly inert surfaces consisting of bioactive molecules that inhibit cell attachment or growth [18, 19]. These techniques involved the design of coatings with different biocidal agents, including antibiotics, quaternary ammonium salts or silver [20–22]. Such compounds may also include organosilanes with active biocidal groups bonded chemically to their chains [23–25]. These can inhibit the growth of microorganisms without releasing toxic products of low molecular mass into the environment. When attached covalently to the surface of a variety of materials, they are able to kill bacteria through contact, and their antimicrobial activity is long-lasting and sustainable. Organosilanes have a minimum of one carbon-silicon bond, for example, Si-CH_3_. This connection is very stable and nonpolar, and in the presence of an alkyl group it causes an increase in low surface energy and its hydrophobic qualities. Many combinations are possible and may contain different types of reactive groups: methoxy, ethoxy, or acetoxy, epoxy, amino, methacryloxy, chloro, or sulphide. Additionally, silanization usually decreases the roughness of native surfaces, and this fact may be behind the increased antiadhesive properties of modified surfaces [24–28].

The aim of this study was to impart antibacterial and anti-adhesive properties of polyethylene (PE) layers after modification with plasma processing and coupling with different active groups. PE materials have been used for many years for the supply of drinking water so biofouling on these materials is a serious problem in water industry. Additionally, PE is a universal polymer which can be used for production of a wide variety of biomaterials, and bacterial biofilms often cause devastating infections and loss of the implanted devices [19]. The native and modified PE surfaces were tested using *A. hydrophila* strain with strong adhesion abilities.

## 2. Materials and Methods

### 2.1. Bacterial Strain

*A. hydrophila *LOCK0968 (GenBank acc. number KC756842) was isolated from the unchlorinated water distribution system in Poland [24, 25]. Bacterial strain was subcultured on antibiotic agar (Merck) slants at 20°C for 3 days and maintained at 15°C. The bacterial cells were grown for 24 hours in 10 mL of buffered peptone water (Merck, Germany) at 20°C without agitation.

*A. hydrophila* cells were grown in the antibiotic broth medium (Merck) at 20°C for 24 hours. The bacterial cells from the liquid cultures were harvested by centrifugation (15 min, 6000 ×g, 5°C), washed twice, and suspended in 100 mL of sterile water to ~9 × 10^8^ CFU/mL by comparison with a McFarland number 3 turbidity standard (Densitometer DEN-1, Grant). Finally, the bacterial suspension was diluted to approximately 9 × 10^2^ CFU/mL.

For the aerobic cultures, 50-fold diluted buffered tryptone water (Merck) with the concentration of 200 mg/L of peptone was prepared and poured (20 mL) into 25 mL Erlenmeyer flasks. After sterilization of the culture medium assuring sterile conditions, the inoculum (2.0 mL) of the bacterial strain and the sterile carrier were added to each flask. The initial cell concentration in the culture medium was 10^1^-10^2^ CFU/mL. The samples were incubated at 15°C on a laboratory shaker (200 rpm) for 2 weeks.

### 2.2. Modification of Carriers

The carriers were prepared in the Polish Academy of Science (PAS) [23–25]. Polyethylene plates (size 60 × 20 mm) were made from granulated Borstar Me 3470-LS BOREALIS used to manufacture the tubes for the transportation of water in water distribution systems. Granulated PE was melted for 3 min and pressed under 100 atm at 180°C to obtain plates which were subjected to modification with the silanes (Table 1). Before carrying out the experiment, the carriers were sterilized in 70% ethanol for 24 h and then by UV-irradiation (*λ* = 265 nm) for 1 h per each side.

**Figure 1:**
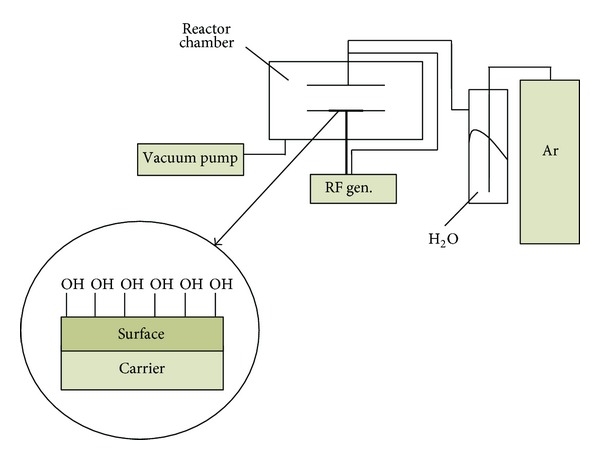
Scheme of plasma generator used for surface activation.

**Figure 2:**
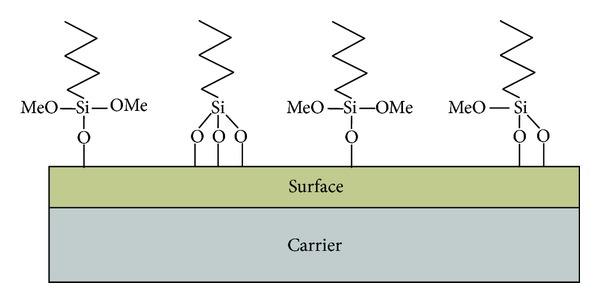
The principle of organosilane attachment to activated surface. Si: silica; O: oxygen; OMe: alkoxy group.

In the beginning of the PE surface modification procedure, the plates were irradiated by radiofrequency generated H_2_O plasma using an apparatus schematically shown in Figure 1. The experimental conditions were as follows: pressure of 300 Pa, power of 40 W, and time of exposure of 2 min. During this stage of the process, a large number of -OH groups (1.5 nmol/cm^2^) were generated on the surface of the carrier. After that, PE plates were immersed into the dry toluene solution (30 mL) containing triethylamine (1 mL) and pyridine (30 *μ*L) placed in two necked 50 mL round bottomed flasks. The flasks were equipped with a dropping funnel connected to them with a pipe through which argon was flowing. The toluene solution (15 mL) of 4-(phenylazo)benzoyl chloride (3.44 × 10^−5^ mol/mL) was being introduced dropwise into a shaken flask for 1 h. The plates were kept in the solution for additional 24 h and then washed with dry toluene and extracted in a Soxhlet apparatus with n-pentane for 5 h. Dried plates were placed in the flask with 10 mL of ammonia hydroxide solution (25%) for 18 h. Then UV absorbance of the solution at *λ* = 324 nm was measured and the concentration of the -OH groups corresponding to the reacted 4-phenylazobenzoyl chloride was calculated. During the second part of the procedure, the activated PE plates were treated with chemical compounds listed in Table 1. The OH-containing plates were immersed for 24 h into 900 mL of the 1,4-dioxane solution of (i) alkoxysilane (7 × 10^−3^ mol) used for the modification and (ii) 2 × 10^−8^ mol of tin(II) octoate as the catalyst. Then, the plates were washed with toluene and dioxane. During the course of chemical reactions, the methoxy or ethoxy goups from the modifying compound were supposed to undergo condensation with hydroxyl groups on the surface (Figure 2).

### 2.3. Determination of Contact Angle and Surface Tension

In order to identify changes that have occurred on the modified surfaces the contact angle measurements for tested materials were taken. Determination of contact angle values for the two different solvents, dimethylformamide (DMF) and water, allowed calculating the surface energy. All measurements were performed using a RAME HART NRL goniometer equipped with a camera CAMERA JVC KYF 70B. The dynamic contact angle was calculated using DROP program and given as average of about 15 measurements. The total surface tension was calculated from the values of the contact angles for two solvents of different polarity (Owens-Wendt's method) [23].

### 2.4. Adhesion and Biofilm Formation

The analysis of bacterial adhesion to the PE carriers was done by three methods, namely, luminometry, plate count, and microscopy. For luminometric tests, the carrier plate was removed from the culture medium, rinsed with sterile distilled water, and swabbed by HY-LiTE 2 sampling pens (Merck) for surface testing. Each pen contained all the reagents necessary in the correct amounts. It is a combined sampling device, test chamber, and reagent dispenser in one. Measurement was done in relative light units (RLU) using a luminometer HY-LiTE 2 (Merck) [24].

The plate count method was used to determine the number of viable cells on the tested surface after incubation. The carrier plate was removed from the culture medium, rinsed with sterile distilled water, and swabbed by a sterile swab for surface testing. The bacterial suspensions were transferred onto PCA medium (Merck) and after incubation (25°C, 48 h) colonies were counted. The number of viable cells on the tested surface was expressed as the log CFU/cm^2^. Adhered bacterial cells were stained with fluorophore, acridine orange, (0.01%; 5 min; filter 490 nm) and observed using a fluorescence microscope (OLYMPUS type BX41) fitted with a 50× lens and with top illumination of the tested surfaces by an external lamp. Images were captured with a digital camera and analyzed using the UTHSCA ImageTool program available on http://compdent.uthscsa.edu/dig/itdesc.html [24, 25, 29].

Mean values were calculated from the data obtained from the three independent experiments. Comparisons between the mean values were performed using the one-way ANOVA test (STATISTICA 10, StatSoft, Poland).

## 3. Results and Discussion

### 3.1. Surface Tension

**Figure 3:**
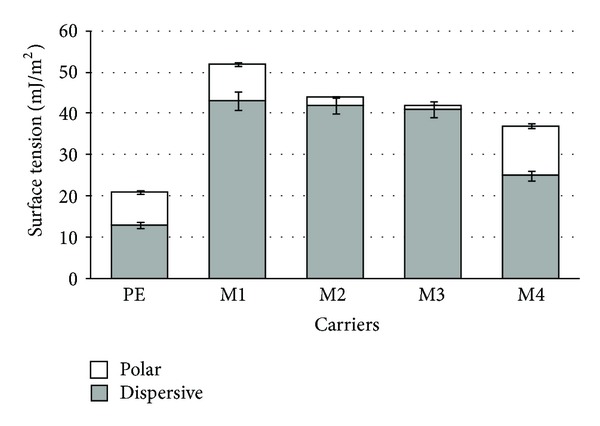
Surface tension for PE surface and its modifications (M1–M4).

Figure 3 presents the results of surface tension measurement for all tested surfaces. The exposure of PE surface to plasma allowed the introduction of its chemical functionalities. We observed that the presence of active groups resulted in a significant increase in the surface energy of the materials tested. In particular, M1 and M4 surface, after chemical modifications with -CH_3_ and -NH_2_ groups, pronounced higher polar forces that contribute to the surface energy. This fact was also evident in other modifications with organosilanes [24, 25].

A generalized relationship between surface tension and the relative amount of adhesion was established as “Baier curve” [16]. It was also found that surfaces with critical surface tension of 20–30 mJ/m^2^ more easily release diverse types of biofouling than materials of higher or lower critical surface tension [30]. All modified materials showed higher values of surface tension in comparison to the native surface. However, it is worth noting that all surfaces become rapidly modified by immersion in natural waters and by adsorption of conditioning films. In our study, the tested surfaces showed various surface tension values, but during the adhesion tests they were under the 14-day contact with water containing a small amount of organic matter, which might have influenced the subsequent adhesive events associated with the attachment of bacterial cells.

### 3.2. Bacterial Adhesion and Biofilm Formation

In the study we used a minimal culture medium (containing 200 mg/L of peptone only), relatively long incubation time (14 days), and an initially very small bacterial concentration in the culture medium (10^1^-10^2^ CFU/mL). These conditions simulated the natural environment in which bacterial attachment and biofilm formation take place.

**Figure 4:**
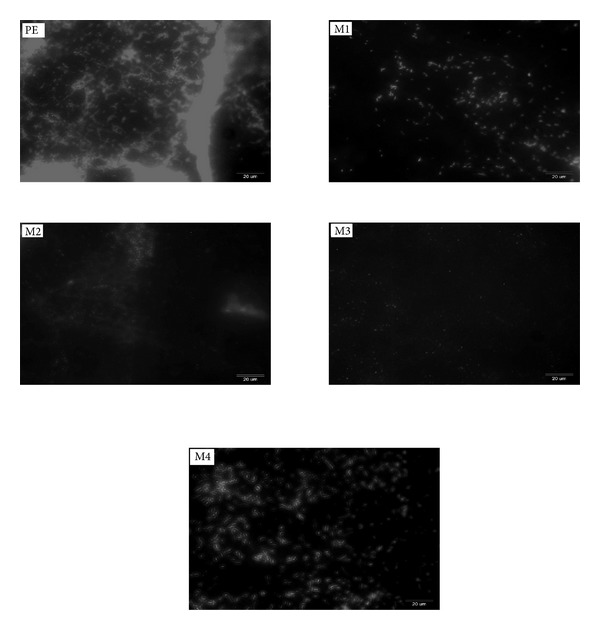
Adhesion of *A. hydrophila* to PE surface and its modifications (M1–M4). Acridine orange staining.

Figure 4 shows exemplary images of the native and modified PE surfaces. The conducted chemical modifications led to a significant reduction in the number of adhered *A. hydrophila* cells. Irregular cell adhesion was detected on the native PE material, resulting in the surface coverage ranging approximately from 10 to 30% of the total area.

**Figure 5:**
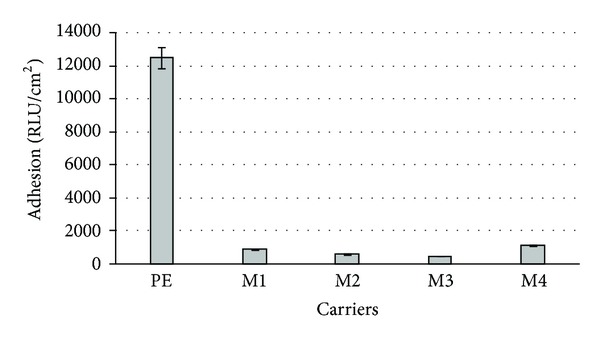
Adhesion of *A. hydrophila *to PE surface and its modifications (M1–M4).

The bacterial attachment to native and modified surfaces was assessed by luminometry and expressed in relative light units per cm^2^ (RLU/cm^2^).  Figure 5 presents the observed changes of the luminescence for tested materials after 2-week incubation period. All the modified surfaces showed lower levels of cell adhesion. For unmodified PE, the maximum value was equal to 12500 RLU/cm^2^. In particular, the modification M3 seemed to have the best antiadhesive features. For this modification the lowest result equal to 467 RLU/cm^2^ was obtained.

**Figure 6:**
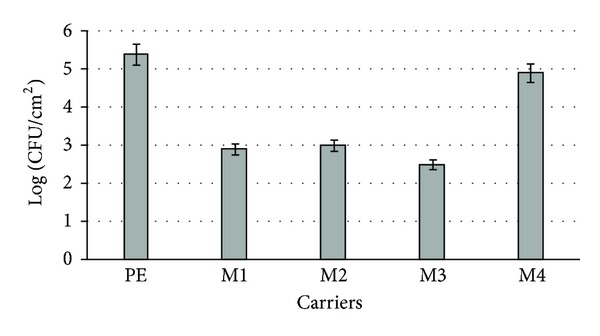
The number of viable *A. hydrophila* cells adhered to PE surface and its modifications (M1–M4).

The number of living bacterial cells on the native surface after two weeks of incubation was 2.5 × 10 ^5^ CFU/cm^2^. The silane modifications led to lowering of the number of living bacterial cells over 1–3 logarithmic units (Figure 6). The significant differences (*P* < 0.05) were found by the ANOVA test in the scope of the adhesion results (RLU/cm^2^ and CFU/cm^2^) for the native PVC and its modified surfaces: M1–M3. The most effective antibacterial modification of PE surface occurred when dimethoxydimethylsilane (M3) was used. Dimethoxydimethylsilane contains two active methoxy groups. It was proposed that these groups are high cytotoxic and play the most important role in inactivation of microorganisms [24].

Generally, biofilm formation on the tested surfaces depended on the chemical type of surface. The inhibition of growth and adhesion of bacteria can be achieved by different chemical compounds covalently binding to the native surface [31–34]. The modification of PE with organosilanes, containing different functional groups, gave the best effects in three cases (M1, M2, and M3). M1–M4 modifications contained reactive methoxy or ethoxy groups, and, additionally, M4 surface possessed functional isobutyl group. According to the literature, methoxysilanes are generally reported to possess higher reactivity and toxicity compared to ethoxysilanes [35]. It is worth noting that, although the molecular structures of M3 and M4 were very similar, the antibacterial performance of M3 was much better than M4. We may assume that antibacterial activity of the compound was also determined by the spatial orientation of molecule. It was confirmed in the previous studies that the molecules that were not identical to the comparator with structural differences, spatial orientation, and/or impurities, might have affected their antibacterial activity [24, 25].

The results obtained by conventional (plate count) and alternative (luminometry) methods were positively correlated (*r* = 0.82), but the correlation was not very strong. This fact may be due to the effect of the antibacterial activity of comparator as well as the arrangement of the bacterial cells on the tested materials. Additionally, the presence of the active groups on the modified surface might result in the stimulation the transition of cells in the “viable but nonculturable” (VBNC) state. The existence of bacteria which survive in the VBNC state is well established and documented [36–40].

According to the literature, a significant number of techniques can be used to measure cell adhesion, but there is no perfect test method [41]. Conventional methods used to determine the number of adherent microorganisms are based on swabbing and plating. In the plate count method, a biofilm is mechanically disrupted, before being serially diluted, and finally colony forming units are determined. Both methods used, the conventional plate count and the unconventional luminometry, require scraping bacterial biofilm. This procedure is difficult because biofilms are resilient, adherent, and quite resistant to stripping by swabs. Additionally, swabbing may give misleading results because of the difficulties in obtaining a homogeneous cell suspension. The use of various procedures supporting “disruption” (chemicals, ultrasound, and laser shockwaves) is usually laborious and may be associated with a reduction in cell viability [41–45]. Therefore, except for classical plate count and luminometry, the fluorescence microscopy as the additional method allowed for accurate evaluation of cell loading.

## 4. Conclusion

Polyethylene as popular biostable material should effectively detach and eliminate the first adhered bacteria. The polyethylene modified with active organosilanes showed desired properties concerning the adhesion and biofilm formation of *A. hydrophila*. Finally, we can conclude that the activation of surface by plasma processing and its chemical modification with active dimethoxydimethylsilane seems promising as suitable coatings for polyethylene as biomedical and water-system material, due to its anti-bacterial and anti-biofouling properties.

## Figures and Tables

**Table 1:** The chemical modifications of polyethylene surface.